# Update on clinical and experimental management of diabetic cardiomyopathy: addressing current and future therapy

**DOI:** 10.3389/fendo.2024.1451100

**Published:** 2024-07-30

**Authors:** Peter Galis, Linda Bartosova, Veronika Farkasova, Monika Bartekova, Kristina Ferenczyova, Tomas Rajtik

**Affiliations:** ^1^ Department of Pharmacology and Toxicology, Faculty of Pharmacy, Comenius University Bratislava, Bratislava, Slovakia; ^2^ Institute for Heart Research, Centre of Experimental Medicine, Slovak Academy of Sciences, Bratislava, Slovakia; ^3^ Institute of Physiology, Faculty of Medicine, Comenius University Bratislava, Bratislava, Slovakia

**Keywords:** diabetic cardiomyopathy, heart failure, diabetes mellitus, diagnostics, pharmacotherapy

## Abstract

Diabetic cardiomyopathy (DCM) is a severe secondary complication of type 2 diabetes mellitus (T2DM) that is diagnosed as a heart disease occurring in the absence of any previous cardiovascular pathology in diabetic patients. Although it is still lacking an exact definition as it combines aspects of both pathologies – T2DM and heart failure, more evidence comes forward that declares DCM as one complex disease that should be treated separately. It is the ambiguous pathological phenotype, symptoms or biomarkers that makes DCM hard to diagnose and screen for its early onset. This re-view provides an updated look on the novel advances in DCM diagnosis and treatment in the experimental and clinical settings. Management of patients with DCM proposes a challenge by itself and we aim to help navigate and advice clinicians with early screening and pharmacotherapy of DCM.

## 1 Introduction

In recent decades, type 2 diabetes mellitus (T2DM) has emerged to become one of the most serious metabolic diseases in the developed world. Incidence of T2DM has skyrocketed as it became a ninth major cause of death. With the incidence of 1 out of 11 adults worldwide it is often described as a pandemic. T2DM, apart from its counterpart type 1 diabetes mellitus (T1DM), is not a congenital disease and is developed during human’s life. A shift from manual work to sedentary lifestyle during 20th century with the combination of longer lifespans both play parts in the emergence of this civilization disease (1).

Although T2DM is usually described as a pathological condition involving high blood glucose (hyperglycemia) and insulin resistance (2), the most severe effects are its complications. Hyperglycemia leads to micro- and macrovascular damage which causes further damage of several organs, most notably nephropathy, neuropathy, retinopathy, and cardiomyopathy (3). Other common complications include diabetic foot, heart attack, stroke, gum disease, sexual dysfunction or cancer (4).

In this review, we focus on diabetic cardiomyopathy (DCM), hence it is considered as one of the most serious secondary complications of T2DM. Type 2 diabetes mellitus has a negative impact on morbidity and mortality of several cardiovascular (CV) diseases and T2DM itself can be a triggering factor of these pathologies and vice versa. On top of that, DCM is usually diagnosed and treated as a separate disease (5). The pathophysiology of DCM consists of cardiac muscle remodeling, hypertrophy (6, 7) and cardiomyocyte cell death (8). However, it has a slightly distinct molecular pathomechanism in contrast with non-diabetic chronic heart failure (HF). There are currently no selective/differentiative diagnostic, nor therapeutic options for these patients.

This review aims to summarize readily available diagnostic and therapeutic tools for cardiologists and diabetologists in the management of DCM patients as well as challenges that current clinical guidelines and practice may pose. Ultimately, we provide an insight into the advances of experimental research and its translation to clinical practice.

## 2 Pathogenesis of DCM

DCM is usually characterized as a contractile dysfunction with later onset of HF without association to dyslipidemia, hypertension or coronary artery disease, but instead caused by metabolic and humoral disruption in connection with hyperglycemia, hyperinsulinemia and insulin resistance (9–11). Despite the great clinical interest towards DCM, its diagnosis and mapping of its distinctive phenotype, the exact pathogenesis of DCM is intricate.

The two main factors that drive DCM are long-term hyperglycemia and insulin resistance. From there it gets complex. The myocardium is metabolically active organ which is highly flexible regarding its sources of energy. It is primarily fueled by free fatty acids (FFA) (70%) and glucose (30%) (12), however, during diabetes there is a reduction in glucose transporters (GLUT-1 and GLUT-4) (13) making FFA the primary source. On one hand, this situation contributes to the lipotoxicity (accumulation of lipid intermediates) and accumulation of reactive oxygen species (ROS), as β-oxidation saturates and fatty acid degradation increases. On the other hand, excessive circulating glucose that could not be employed in myocardium leads to generation of advanced glycation end products (AGEs) through the hexosamine and polyol protein kinase C (PKC)-activating pathways (12–14). AGEs propose a threat as they induce protein structural changes, collagen cross-links, exacerbates oxidative stress and inflammation, activate PKC and cause irreversible damage to hearts microvasculature (15, 16). Long-term hyperglycemia therefore leads to chronically increased oxidative stress in cardiomyocytes which can cause mitochondrial and DNA damage, lipid peroxidation in the cell membrane and activation of different forms of cell death (17).

Myocardial apoptosis was in fact found to be 85 times more pronounced in diabetic hearts than in non-diabetic (18, 19). Moreover, accumulating evidence has shown that iron-mediated ferroptosis plays significant part in DCM. Main driving factor of ferroptosis is intracellular accumulation of Fe^2+^ which oversaturates lipid ROS synthesis while antioxidant pathways are inhibited (20). Furthermore, necroptosis, which is another form of programmed necrotic cell death, could be mediated in receptor-interacting protein kinase 3 (RIPK3)-dependent manner upon activation of Ca^2+^/calmodulin-dependent protein kinase II (CaMKII) in diabetic mice (21). Knockout of RIPK3 leads to decreased activation of CaMKII and to improvement of cardiac function, as well as decreased extent of necroptosis. This is particularly interesting because it has been shown that CaMKII can be upregulated in hyperglycemic conditions and it worsens the heart function leading to arrythmias or pathological remodeling (22, 23). Therefore, a possible future therapy targeting CaMKII activity in heart could be beneficial also for the T2DM and progression of DCM. Pyroptosis, a proinflammatory type of PCD (24), is also involved in the pathogenesis of the DCM and is the least studied one. Actual evidence regarding pyroptosis in DCM encompass activation of NLRP3 (NOD-like receptor 3) and AIM2 (Absent in Melanoma protein 2) inflammasome. It has been suggested that high glucose conditions promote activation of NLPR3 via nuclear factor kappa-light-chain-enhancer of activated B cells (NF-κB) signaling (25) which in turn activates caspase-1, interleukin-1β (IL-1β) and K^+^ efflux (26). Moreover, activation of NLPR3 promotes the TGF-β/Smad remodeling pathway leading to fibrosis in DCM even under conditions when inflammasome is not activated (27).

Chronic myocardial inflammatory status is another factor that contributes to DCM. T2DM-associated metabolic disorders (hyperglycemia, hyperlipidemia) can directly induce cytokine expression and release from cardiac cells (28, 29). Moreover, these metabolic derangements can activate systemic and cardiac inflammatory cells, which infiltrate and accumulate at the site of cardiac fibrotic lesions. These cells secrete cytokines and chemokines such as tumor necrosis factor (TNF) (28); interleukins, such as IL-6 (30), IL-1β, TGF-β (31); and interferon-γ (32) that can induce or aggravate cardiac hypertrophy and remodeling. Additionally, these proinflammatory agents are involved in the development of rapid cardiac contractile dysfunction (33, 34). Diabetes-related chronic inflammatory state can therefore produce progressive qualitative damage of myocardial tissue resulting in progressive left ventricular, either systolic or diastolic, functional impairment.

Calcium handling is another aspect that is altered by DCM. Not only is it crucial for excitation-contraction coupling and maintaining cardiomyocyte homeostasis, but its dysregulation also plays role in maladaptive changes that might lead to cardiomyocyte cell death. DCM hinders the activity of multiple Ca^2+^-handling proteins, including L-type Ca^2+^ channels or sarcoendoplasmic reticulum Ca^2+^ ATPase (SERCA), as well as promotes inflammation, oxidative stress and mitochondrial damage (35).

However, diabetes driven effects on heart should not be limited on myocardium only. Coronary circulation is crucial in maintaining blood flow even under pathological states like vessel occlusion or stenosis (36). Micro-/macroangiopathies are well described vessel-targeting phenomena when it comes to diabetic complications (37) and the coronary vessels seem to be no exception. Insulin resistance can generally lead to endothelial dysfunction as high level of ROS hinders NO synthesis. This in turn results in protein kinase G (PKG) downregulation and promotes cardiac hypertrophy (38).

These diverse mechanisms are some of the hidden players responsible for the onset of cardiac remodeling which most frequently manifests as early-on asymptomatic diastolic dysfunction that later progresses into more severe pathological phenotype (9).

### 2.1 Remodeling and fibrosis in DCM

Long-term T2DM promotes proliferation of cardiac fibroblasts leading to interstitial extracellular matrix (ECM) deposition and myocardial interstitial fibrosis which contributes to LV anatomic and functional remodeling (39). The consequences of these maladaptive changes are increase in myocardial stiffness and reduced ventricular compliance which play a vital role in the development of LV diastolic dysfunction leading to heart failure with preserved ejection fraction (HFpEF), the most common form of HF in T2DM (40). Numerous humoral factors have already been proved to contribute to the pathomechanism of DCM. The best known include activation of the renin-angiotensin-aldosterone system (RAAS), transforming growth factor-β (TGF-β) signaling and advanced glycation end-products, among others.

A number of studies have shown that the RAAS is closely related to myocardial hypertrophy and fibrosis in DCM (6, 7). Increased angiotensin II (Ang II) levels stimulate angiotensin receptor-1 (AT_1_R) acting directly on cardiac fibroblasts, increasing collagen synthesis and decreasing collagen decomposition, resulting in cardiac hypertrophy and fibrosis (41, 42). Myocardial inflammation in diabetic heart is also mediated by the RAAS and thus promotes cardiac remodeling (7). These changes manifest as reduced ventricular compliance and cardiac systolic and diastolic dysfunction (41, 42).

It is suggested that TGF-β signaling is required for Ang II to induce both cardiac hypertrophy and fibrosis (43). TGF-β is an essential regulator of cardiac fibroblast proliferation and differentiation that contributes to remodeling in DCM. TGF-β1 exerts a strong profibrotic effect on cardiac fibroblasts, myofibroblasts, and other cardiac cells, causing the progression of myocardial fibrosis (40, 44). The TGF-β profibrotic signal in DCM may be due to the direct action of high glucose on TGF-β secretion and activation (34, 45), due to accumulation of AGEs (46), through Ang II pathway activation (47), or as a consequence of changes in the expression of some microRNAs (48). Ultimately, activated TGF-β in the myocardial tissue causes abnormal ECM accumulation, leading to cardiac fibrosis in diabetic heart.

Apart from RAAS or TGF-β, AGEs are specific for various complication of T2DM, including atherosclerosis, heart remodeling and fibrosis in DCM. High glucose levels result in non-enzymatic addition-elimination reaction of sugar carbonyl groups with free amino acids of proteins (49). These defective proteins exert multiple negative effects on vascular endothelial cells and cardiomyocytes, including impairment of collagen degradation by matrix metalloproteinases (MMPs) and cause collagen cross-links. This leads to the loss of collagen elasticity and degradation, increase in cardiac interstitial and perivascular fibrosis and subsequently to a reduction of arterial and myocardial compliance (50, 51). Moreover, AGEs bind to their receptors (RAGE) which are overexpressed in DCM. Upon the activation of RAGE, excessive ROS formation and increased mitogen-activated protein kinase (MAPK) signaling takes place, further enhancing fibrosis, macrophage adhesion and inflammation and ultimately leading to atherosclerosis and cardiac remodeling (15, 16, 52, 53).

### 2.2 Metabolic impairment leading to DCM

There is a strong animal and clinical evidence that development of T2DM is correlated with low thyroid function. People with lower triiodothyronine (T3) levels have a greater risk of increased blood glucose, impaired insulin sensitivity and T2DM development (54–57). Conversely, patients treated with thyroid hormones gradually improve their glycemic control (57). Low T3 level is not an exclusive risk factor for T2DM although it has been observed in HF patients. Low T3 or T3/T4 ratio inversely correlated with HF progression (NYHA classification) and NT-proBNP serum levels (58–60).

T3 impacts heart after acute injury as a fetal-like feature to induce proliferation and tissue repair. However, myocardium has a very limited regenerative potential and undergoes remodeling that can temporally compensate for the contractile dysfunction, until it is met with low energy profile and becomes maladaptive which leads to HF. Therefore, after stress stimuli diminish, a drop in T3 serum levels takes place (61). This drop of T3 levels affects BNP serum levels and, as it is experimentally suggested, has an impact on microvascular blood flow (MBF). Clinically speaking, impaired MBF is a sign of ischemic heart disease, idiopathic dilated cardiomyopathy, DCM and ultimately HF (62).

Plenty of experimental evidence support these hypotheses. For instance, low dose T3 treatment improved contractility in streptozocin (STZ)-induced rat model of T2DM (63). In genetic model of DCM in Syrian hamsters, mixture of T3 and T4 deceased loss of cardiomyocytes mediated by improved MBF (64). Additionally, observational studies found a significant association of low T3 with high mortality in hospitalized patients with HF (65). Unfortunately, current guidelines for the management of T2DM, HF or DCM does not include thyroid hormone diagnostic screening and/or the therapy.

## 3 Novel trends in diagnosing DCM

### 3.1 Current approaches and challenges in diagnostics of DCM

Diagnosing DCM alone poses a clinical challenge due to potential symptom and imaging overlap with other heart diseases, particularly chronic HFpEF (66). The progression of DCM is often gradual, spanning years or even decades, driven by chronic exposure to metabolic abnormalities associated with diabetes (67). This gradual onset makes pinpointing DCM difficult and can lead to delayed diagnosis. Additionally, diabetic cardiomyopathy can present with subtle or asymptomatic changes in cardiac structure and function before overt HF symptoms emerge. This is in large part the cause of missed early detection without routine screening or specialized diagnostic tests (68). Unlike the other types of HF, there are currently no specific diagnostic criteria or biomarkers exclusively for DCM. Diagnosis relies on integrating clinical evaluation, imaging studies, and biomarker analysis which may lack specificity for this condition (69).

Therefore, a comprehensive evaluation is crucial for accurate diagnosis and appropriate management. Although no approved therapeutic strategies exist to treat or prevent progression of stage B DCM, ongoing efforts target various pathological mechanisms. Recent CV outcome trials (CVOTs) have identified newer therapeutic agents with CV benefits, such as sodium-glucose cotransporter-2 (SGLT-2) inhibitors reducing hospitalization for HF and glucagon-like peptide-1 (GLP-1) receptor agonists reducing major adverse CV events (MACE), albeit with inconsistent effects on HF outcomes (70). Clinical practice guidelines recommend screening high-risk patients for HF (71).

Diagnosing DCM typically involves a combination of clinical assessment, imaging techniques, like echocardiography and magnetic resonance imaging (MRI) for diastolic function and ventricular mass assessment, or biomarker analysis (72). Exclusion of coronary artery, valvular or congenital heart disease is possible using coronary angiography and Doppler echocardiography imaging. Endomyocardial biopsy is indicated for infiltrative heart disease concerns. Magnetic resonance (MR) spectroscopy, measuring myocardial triglyceride content, is an emerging research tool for DCM diagnosis and pathogenesis (73).

Addressing these challenges necessitates a comprehensive CV assessment in diabetic patients, including routine cardiac dysfunction screening, awareness of DCM’s unique features and utilization of advanced imaging modalities and biomarkers for early detection and risk stratification. Collaborative efforts between cardiologists, endocrinologists and primary care providers are essential for optimalization of DCM diagnosis and management. Ongoing research into novel markers and techniques holds promise for future progress in this field.

### 3.2 Novel insights on the biomarkers of DCM

**Figure 1:**
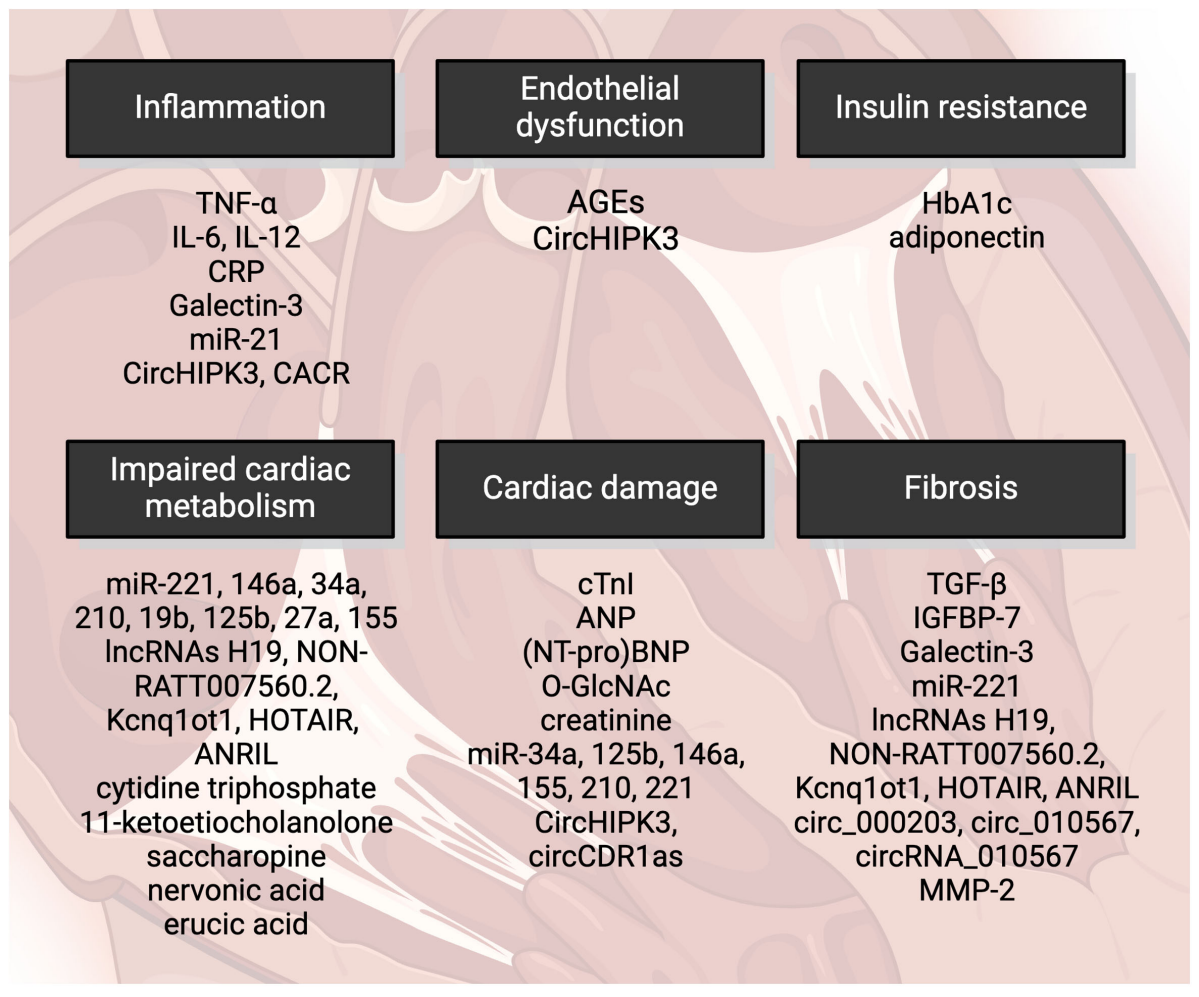
Markers of diabetic cardiomyopathy include cardiac damage characterized by contractile dysfunction and cell death. Impaired cardiac metabolism is also a feature, with oxidative stress playing a significant role. AGEs, advanced glycation end-products; HbA1c, glycated hemoglobin; miRNA, micro RNA; lncRNAs, long noncoding RNAs; TGF-β, transforming growth factor β; IGFBP-7, insulin-like growth factor binding protein-7; MMP-2, matrix metalloproteinase-2; cTnI, cardiac troponin I; NT-proBNP, N-terminus of prohormone brain-derived peptide; O-GlcNAc, O-linked N-acetylglucosamine; TNF-α, tumor necrosis factor α; IL, interleukin; CRP, C-reactive protein. Created by BioRender.com.

In contemporary clinical practice, there is a notable lack of specific markers for dilated cardiomyopathy. This deficiency arises from two primary challenges: First, the identification of a definitive marker for DCM is inherently difficult. Second, the relevance of such markers has yet to be established in routine clinical practice. Currently, asymptomatic patients with T2DM are monitored for HF risk using standard HF markers, while HF patients—and indeed all patients under medical care—are routinely assessed for metabolic disturbances in a reciprocal manner. However, identifying early and highly sensitive diagnostic as well as prognostic biomarkers is very important for preventing the development of DCM and improving the prognosis of DCM patients. Novel biomarkers could also provide a basis for discovering new targeted interventions for DCM. Graphical illustration of markers of DCM are depicted in **Figure 1**. Here, we present an array of well-established markers for T2DM and heart failure that are currently used in clinical practice for patients with DCM. Additionally, we introduce new and experimental markers that are under investigation.

#### 3.2.1 Glycated hemoglobin

It has been documented that serum glycated hemoglobin (HbA1c) levels are significantly higher in individuals with DCM in comparison to those with T2DM alone (9). However, higher HbA1c levels in patients with T2DM increase their risk of developing HF but paradoxically in T2DM patients with already developed HF are higher HbA1c levels associated with increased survival in the 2-year horizon (74). The use of HbA1c as a potential prognostic marker in patients with DCM is therefore still ambiguous and requires further investigation. Moreover, the impact of various medications on glycemic control in studied patients cannot be underestimated.

#### 3.2.2 Cardiac troponin I

Observational studies (75, 76) have found elevated cardiac troponin I (cTnI) as a marker of cardiac damage in asymptomatic diabetic patients. cTnI is considered one of the current clinical markers of cardiac damage in diabetic patients, alongside C-reactive protein (CRP) and electrocardiography (5). cTnI as a general marker of cardiac damage is not specific for high glucose- or T2DM-induced cardiac injury and thus cannot be considered a specific marker of DCM. However, cTnI presents still a reliable diagnostic tool for monitoring asymptomatic T2DM patients for the development of HF risk.

#### 3.2.3 Natriuretic peptides

Natriuretic peptides including atrial natriuretic peptide (ANP) and brain natriuretic peptide (BNP) were proposed as potential biomarkers of DCM before (9, 11, 77). Particularly BNP could be an inexpensive and easily attainable marker of preclinical ventricular diastolic dysfunction in T2DM patients (78). Recent studies analyzing cohorts of T2DM patients suggested N-terminal of prohormone BNP (NT-proBNP) as a relevant biomarker that could help detect HF in T2DM (79, 80). On the other hand, in T1DM patients, ANP but not BNP appears to be a sensitive biomarker for early diastolic dysfunction (81).

#### 3.2.4 O-linked N-acetylglucosamine

Increased O-linked N-acetylglucosamine (O-GlcNAc) levels were previously linked to the adverse cardiac effects of T2DM, including impaired contractility, calcium handling and abnormal stress response (82). O-GlcNAcylation was further implicated in the development of CV dysfunction in T2DM (83). Maladaptive O-GlcNAc protein modification serves as a critical regulator of the diabetic phenotype in human hearts. Thus, restoring physiological O-GlcNAc balance in the heart may also represent a novel therapeutic approach for T2DM-induced HF (84).

#### 3.2.5 Inflammatory, fibrotic and antioxidant markers

Serum inflammatory mediators such as TNF-α, interleukins and C-reactive protein (CRP), elevated fibrotic markers like TGF-β and insulin-like growth factor binding protein-7 (IGFBP-7) and decreased antioxidant markers adiponectin and leptin are suggested to uncover early onset of DCM (85). Elevated levels of IL-12/23p40 were proposed as a potential indicators of disrupted heart rate variability in T2DM (86). A panel of AGEs, IL-6, TNF-α, insulin and creatinine might be used for early detection of diastolic dysfunction in T2DM (87). Two fibrotic markers, transforming growth factor-beta (TGF-β) and insulin-like growth factor-binding protein 7 (IGFBP-7), are significantly elevated in patients with diastolic dysfunction. Specifically, T2DM patients with diastolic dysfunction exhibit higher levels of TGF-β and IGFBP-7 compared to non-diabetic patients with diastolic dysfunction. Additionally, T2DM patients without diastolic dysfunction show increased levels of these markers when compared to healthy controls. This suggests that TGF-β and IGFBP-7 may serve as markers for early heart dysfunction risk in asymptomatic T2DM patients (85). Very recently, several new potential biomarkers for DCM have emerged, including MMP-2 for detecting early fibrosis in DCM (88).

Several other novel markers for DCM have been proposed by recent studies, including galectin-3 (Gal-3) and adiponectin (85, 89, 90). Gal-3, a chimeric galactose-lectin family protein, is strongly linked to cardiac fibrosis and inflammation. Since cardiac fibrosis plays the key role in the development of DCM, Gal-3 can be involved in the progression of DCM through several different mechanisms. Gal-3 levels were found to be a good predictor of HFpEF in T2DM patients (91) and additionally, elevated Gal-3 were observed in T2DM patients compared to non-diabetic ones (92). This makes galectin-3 (Gal-3) a promising candidate for diagnosing the potential early onset of cardiomyopathy in T2DM patients.

Adiponectin is a member of adipokines that affect insulin sensitivity and hyperadiponectinemia is considered an independent risk factor for DCM (93). Low adiponectin levels in adolescents with T2DM correlated with lower cardiac circumferential strain. Since abnormal circumferential strain may represent the earliest evidence of functional cardiac impairment in T2DM, adiponectin may serve as an early biomarker of functional changes in T2DM (89). In T2DM patients with acute coronary syndrome, high adiponectin was associated with increased risk of hospitalization for HF, death from CV causes and all-cause mortality (94).

#### 3.2.6 Noncoding RNAs

New evidence also suggests several microRNAs (miRNAs) and long non-coding RNAs (lncRNAs) as biomarkers of DCM (95), especially those associated with cardiac inflammation (miRNA-21) and redox signaling (miRNA-221, miRNA-146a, miRNA-34a, miRNA-210, miRNA-19b, miRNA-125b, miRNA-27a, miRNA-155) as well as with cardiac hypertrophy (miRNA-221) and apoptosis (miRNA-34a, miRNA-125b, miRNA-146a, miRNA-155, miRNA-210, miRNA-221) (95). LncRNAs may also participate in the development of cardiac hypertrophy and HF in DCM via regulating redox and inflammatory signaling. Particularly lncRNAs H19 (96), NON-RATT007560.2 (97), Kcnq1ot1 (98), HOTAIR (99) and ANRIL (100) associated with cardiac remodeling in DCM via regulating cardiomyocyte apoptosis and oxidative stress. Finally, several circular RNAs (circRNAs) involved in the progression of DCM including circ_000203, circRNA_010567, circHIPK3, CACR and circCDR1as were discussed in correlation to DCM (101).

#### 3.2.7 Metabolic markers

Some plasma biomarkers of abnormal metabolism have been recently identified using liquid chromatography-mass spectrometry (LC-MS)-based metabolomics (102). Among selected metabolites, cytidine triphosphate (associated with pyrimidine metabolism), 11-ketoetiocholanolone (metabolite of cortisol/steroid hormone biosynthesis), saccharopine (metabolite of lysine), nervonic acid (precursor of myelinization), and erucic acid (inhibition of mitochondrial oxidation of other fatty acids, particularly in cardiac tissues), have emerged as promising candidates for differential diagnostics between T2DM and DCM. The precise role of nervonic and erucic acids in HF are not yet fully understood, although they may function as compensatory mechanism in reducing the accumulation of very long-chain fatty acids (VLCFA), which trigger various pathological processes such as mitochondrial dysfunction, oxidative stress, and inflammation (103). Saccharopine’s involvement in the disruption of normal mitochondrial function has been documented (104) but has not been studied in failing hearts. The role of cytidine triphosphate and 11-ketoetiocholanolone in DCM remain elusive, necessitating further investigation.

## 4 Current therapeutic approaches to mitigate the symptoms of diabetic cardiomyopathy

**Figure 2:**
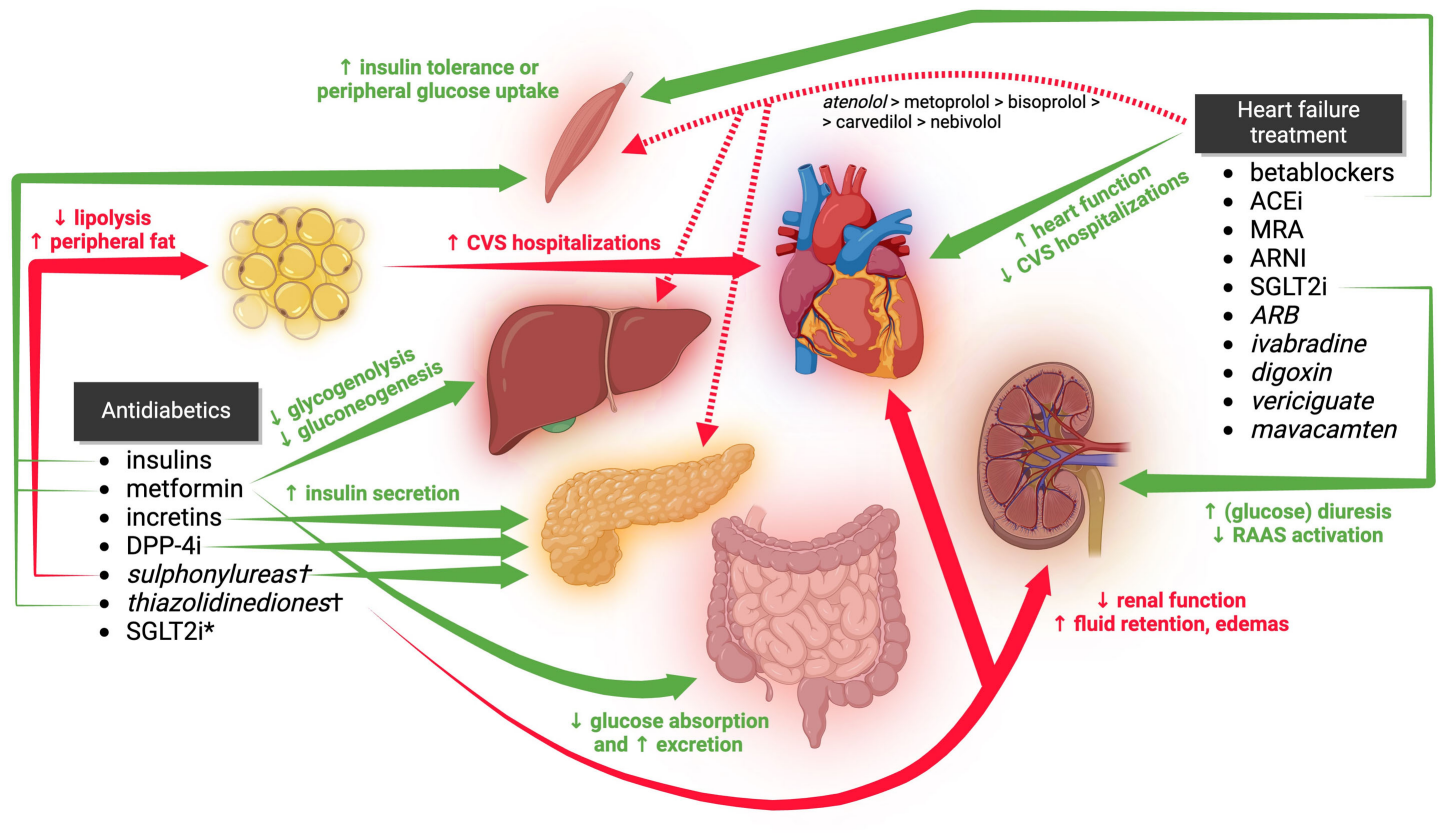
The clinical effects of antidiabetic and anti-heart failure medications on glucose tolerance, cardiac function, and hospitalization rates in DCM are illustrated in the figure. However, certain secondary and tertiary effects are not depicted. Medications listed in italics are not considered first-line treatments. Those marked with a † are either not recommended or contraindicated in DCM. CVS, cardiovascular; ACEi, angiotensin-converting enzyme inhibitors; MRA, mineralocorticoid receptor antagonists; ARNI, angiotensin receptor blocker and neprilysin inhibitor; SGLT2i, sodium-glucose co-transporter 2 inhibitors; SGLT2i*, effects of SGLT2i on glycemia are shown within HF treatment; ARB, angiotensin receptor blockers; DPP-4i, dipeptidyl peptidase-4 inhibitors. Created by BioRender.com.

To this date, there is no specific therapy for DCM. The treatment consists of pharmacotherapy alongside lifestyle modifications. The pharmacological treatment of DCM is currently based on lowering high blood glucose and mitigation of failing heart (**Figure 2**). Other therapeutic agents such as antihyperlipidemics, diuretics or anti-aggregants/anti-coagulants are commonly prescribed as an adjuvant therapy.

### 4.1 Antidiabetic drugs

As mentioned in preceding paragraphs, hyperglycemia is a risk factor for CV morbidity and increases the risk of hospitalization in patients with DCM (39, 40). Therefore, various antidiabetic drugs are used to prevent hyperglycemia from damaging blood vessels, kidneys and ultimately the heart. It is important to clarify that antihyperglycemic effect of antidiabetic agents alone does not necessarily correlate with efficacy or safety in patients with DCM. Several molecules seem to be beneficial; others have no significant effect, and some may even increase the risk for hospitalization. These clinical findings suggest that specific antidiabetic drugs may influence DCM beyond their glucose-lowering effect. It has been hypothesized that it can be, at least partially, explained by their effect on body mass – newer antidiabetics with anti-obesity activity have lower risk for hospitalization than those ones increasing the body mass. Nevertheless, no direct scientific evidence has been published yet.

Here we provide a list of medications commonly prescribed for T2DM treatment. Our findings confer with current (year 2023) practical guidelines of European Society of Cardiology (ESC) for the management of CV disease in patients with diabetes (5) and are also supported by several other independent sources. American Heart Association/American College of Cardiology/Heart Failure Society of America (AHA/ACC/HFSA) have not published such guideline yet. Instead, their Guideline for the Management of Heart Failure (105) provide a list of medication deleterious for HF prognosis, including several agents used to treat T2DM. On the other hand, this document is short with the information for how to treat HF patients with T2DM, stating only the role of SGLT2 inhibitors (SGLT2i).

#### 4.1.1 Glucagon-like peptide-1 receptor agonists

GLP-1 receptor agonists, or simply incretins, are a relatively novel class of antidiabetics. The class consists of several synthetic subcutaneously administered GLP-1 analogues such as shorter acting exenatide, liraglutide, lixisenatide, longer acting dulaglutide, albiglutide, efpeglenatide and semaglutide and a dual GIP/GLP-1 agonist tirzepatide (106). Their antihyperglycemic effect is provided by the stimulation of insulin release, while they concurrently inhibit excessive glucagon release. On the top of that, they possess a significant anti-obesity activity by inducing anorexia (appetite suppression) and they also help to slow down gastric emptying (107).

While they are known to be effective antidiabetic drugs, their effects on CV morbidity and mortality are studied in a lesser extent, unlike exenatide or liraglutide which are among the most studied molecules. Only a handful of small trials have been conducted on these molecules with slightly beneficial or neutral effect on HF hospitalizations overall. On the other hand, they were linked with a tachycardia, which raises a concern about their beneficial effects on failing hearts (5, 107, 108). Ultimately, more comprehensive trials including newer molecules (e.g., dulaglutide or semaglutide) are missing. Therefore, 2023 ESC guidelines mark GLP-1 agonists as a second line therapy (therapy for consideration) for HF-related outcome in patients with T2DM (5).

#### 4.1.2 Dipeptidyl peptidase-4 inhibitors

Dipeptidyl peptidase-4 (DPP4) is an enzyme degrading incretins, thus DPP4 inhibitors increase the activity of natural incretins, somewhat mimicking the effects of GLP-1 agonists (although not all). Current DPP4 inhibitors include sitagliptin, vildagliptin, linagliptin, saxagliptin and alogliptin (109). Gliptins have been studied even less thoroughly compared to incretins in the case of CVOTs. Some small studies found inconclusive beneficial and detrimental effects on the rate of hospitalizations. Larger CVOTs with sitagliptin and linagliptin found a neutral effect. Saxagliptin and alogliptin were more prone to increasing risk of HF hospitalizations. Vildagliptin was the least studied member of this class with no promising results (5, 105, 107, 108). These inconclusive findings might be a result of baseline differences in the use of metformin, thiazolidinediones and insulin which also affect HF risk.

In conclusion, sitagliptin and linagliptin can be considered for antihyperglycemic therapy in HF patients, saxagliptin and alogliptin should be avoided (5, 105).

#### 4.1.3 Insulins

Insulins are used in patients with unsatisfactory treatment with oral antidiabetics. These patients are considered to have worse CV outcomes (110). Two larger randomized trials [ORIGIN (111) and DEVOTE (112)] demonstrated that longer-acting basal insulins (glargine and deglutec) have at least neutral effects on HF hospitalizations. These two insulins can be considered in patients with DCM (5).

#### 4.1.4 Metformin

Metformin, as a single clinically used drug of biguanide group, has been used as first-line therapy of T2DM. In the past, metformin was contraindicated in diabetic patients with HF due to a possible lactic acidosis. However, this contraindication was later on removed (113, 114) as the frequency has been proved to be very rare – less than 1 in 10, 000 patients according to the summary of product characteristics (SPC) (115). Some sources (116) claim a higher frequency of 1-10 in 10,000. Nowadays, metformin is considered to be a safe option for patients with DCM with better clinical outcome of HF than sulphonylureas or insulins (5). Nevertheless, it is still contraindicated in decompensated HF due to the high risk of lactic acidosis (114) and its particular outcome on HF hospitalizations, due to lack of sufficient CVOTs, remains unclear.

#### 4.1.5 Sulphonylureas

Sulfonylureas are a class of oral antidiabetics that stimulate the release of insulin from pancreatic β cells. Despite being effective antidiabetics in lowering high blood glucose and insulin resistance, they increase the risk of hypoglycemia and body weight gain (117) which are risk factors for HF. Clinical evidence for their role in CV events is inconsistent. Earlier agents with higher risk of hypoglycemia and body weight gain (such as 1st generation tolbutamide and with a lesser extent 2nd generation glibenclamide, gliclazide or glipizide) tend to be increasing the risk for the HF hospitalizations. On the contrary, newer agents (mostly 3rd generation glimepiride) seem to have a safer profile in the case of HF complications (5, 107, 108). Nevertheless, sulphonylureas as a whole group are not recommended for the use in diabetic patients with HF according to the current guidelines (5).

#### 4.1.6 Thiazolidinediones

Thiazolidinediones are a class of oral antidiabetics acting on intracellular peroxisome proliferator-activated receptor-γ (PPR-γ) which in turn stimulate expression of the genes leading to increase of insulin sensitivity, improving lipid metabolism and other CV effects (118). In spite of these beneficial properties, both pioglitazone (119) and rosiglitazone (120–122) increase fluid retention, edemas (due to the decrease in renal filtration and diuresis) and body mass gain. Thiazolidinediones were shown to significantly increase (by over two-fold) the risk of HF hospitalizations in several clinical trials (119–122) and therefore are considered contraindicated in all HF patients with or without diabetes (5, 105).

#### 4.1.7 Sodium–glucose co-transporter-2 inhibitors

Sodium–glucose co-transporter-2 inhibitors are a novel class of oral antidiabetics with characteristic properties. The members include empagliflozin, dapagliflozin, canagliflozin, sotagliflozin and newer ertugliflozin. They increase renal glucose excretion which in turn lowers glycemia (123). SGLT2i also decrease insulin resistance, blood pressure and body mass, while not causing hypoglycemia (124). However, in combination with other antidiabetics they can increase the risk of hypoglycemia.

SGLT2i are the first class of antidiabetics with widely proven clinical benefits on HF prognosis (5, 105).

### 4.2 Heart failure drugs

According to the current guidelines of ECS (5), diabetic patients with HF are treated with standard therapy as patients without diabetes. Importantly, diabetic patients with HF have higher CV risk.

The standard 1st line therapy for HF includes quadruple combination of SGLT2 inhibitor, ACEi (angiotensin converting enzyme inhibitor)/ARNI (angiotensin receptor–neprilysin inhibitor), betablocker and MRA (mineralocorticoid receptor antagonist). Their efficacy has been proven in numbers of clinical trials with positive impact on the rate of hospitalizations a survival in patients with or without diabetes. The cardioprotection of this combination includes improved cardiac contractility with partially attenuated myocardial fibrosis, inflammation, remodeling and decreased afterload and preload via several molecular mechanisms (5, 71, 105). While these medications are commonly used in patients with heart failure with reduced ejection fraction (HFrEF), they have not been specifically clinically tested in patients with T2DM, except for SGLT2 inhibitors. Furthermore, their efficacy has not been specifically evaluated for DCM.

As 2nd line therapy, ivabradine, vericiguat or ARB (angiotensin receptor blocker) can be chosen in patients with insufficient response to the treatment or in the case of adverse effects of 1st line medications (5, 125). Data for DCM regarding the drugs considered first-line treatments is even more limited.

The most interesting therapeutic group used in DCM are SGLT2i. It’s the only therapeutic class with strong clinical evidence of both antihyperglycemic and cardioprotective properties (126), although they were initially developed as antihyperglycemic drugs. Two of them, namely empagliflozin and dapagliflozin, were later repurposed for the treatment of HF in the presence or without the presence of diabetes (5, 125). Canagliflozin has been successful in trials as well (127), however, it is not officially indicated for HF. Another SGLT2 inhibitor, sotagliflozin, has shown some positive effects on HF (128) although, it is available for the US market only. It was withdrawn from the European Union in 2022 at the request of the marketing-authorization holder (129).

SGLT2i exert a complex cardioprotective mechanism independent from their antihyperglycemic effects. The inhibition of SGLT2 leads to decreased Na^+^ reabsorption, thus increased Na^+^ concentration in distal tubule which enhances diuresis. This simple change in Na^+^ homeostasis leads to enhanced tubuloglomerular feedback mechanism, decreased intraglomerular pressure and decreased heart preload and afterload (due to the diuresis). Decreased intraglomerular pressure presumably attenuates the activation of sympathetic and renin-angiotensin-aldosterone system, thus leading to the amelioration of cardiac remodeling and inflammation, improved diastolic function, together with improved renal function. These ‘off-target’ mechanisms also include improvement in hematocrit, blood pressure and body weight which can all contribute to positive renal and heart effects (130–134).

#### 4.2.1 ACE inhibitors

ACE inhibitors have been found to be beneficial in improving insulin sensitivity and glycemic control (135–138). The proposed mechanism behind this follows decreased bradykinin metabolism and its increased plasma levels, activation of soluble guanylate cyclase (sGC) upon bradykinin B_2_ receptor activation with subsequent nitric oxide release and vasodilatation leading to the increased GLUT4 translocation in peripheral tissues (139).

#### 4.2.2 Betablockers

There are 4 betablockers currently indicated for the chronic HF treatment: 2nd generation (β_1_-selective) metoprolol (140) and bisoprolol (141); 3rd generation (with vasodilatory effect) carvedilol (142) and nebivolol (143). Moreover, several smaller studies have explored the use of atenolol and labetalol, which are currently considered off-label in HF treatment. The use of atenolol has been associated with positive outcomes on hemodynamic functions (144–146), although inferior to nebivolol (144, 145). Data for labetalol use in HF is lacking some evidence, although it may be considered at least safe in HF patients (147).

We understand that betablockers are effective in treatment of chronic HF, however, the official ESC guidelines for the management of HF patients with T2DM (5) do not explicitly address their effects on glycemic control and that is what we aimed for in the following paragraphs.

1st generation non-selective betablockers block both β_1_ and β_2_ receptors, thus decreasing insulin secretion, glycolysis and lipolysis, while increasing glycogenolysis and gluconeogenesis and are prone to mask the symptoms of hypoglycemia (148, 149). 1st generation betablockers (such as propranolol) also decrease peripheral vasodilatation (150). On the other hand, they also affect glucagon release (151) with increased risk of hypoglycemia (152). These effects are slightly improved within the 2nd generation (153), although they are still linked with increased glycemia and HbA1c. Metoprolol displays more negative metabolic effects compared to metabolic-neutral bisoprolol (154), due to its higher β_1_-selectivity. 3rd generation vasodilatory carvedilol (155) and nebivolol (145) provide better results, while nebivolol seems to be superior (156).

Likewise in ACE inhibitors, vasodilation is connected to the increased insulin sensitivity and better glycemic control (157). Despite the negative metabolic effects, clinical trials proved that betablockers are beneficial in all HF patients with or without T2DM (140). Official guidelines don’t prefer any particular betablocker over the others in the case of T2DM patients with HF (5), however, based on the available data, most notably nebivolol, carvedilol or bisoprolol are preferable for HF patients with concomitant T2DM. Metoprolol and atenolol should be considered only as 2nd or 3rd line, respectively. Labetalol lacks the necessary data for this treatment setup, although it possesses a theoretically favorable pharmacological profile for HF patients with T2DM.

#### 4.2.3 Mineralocorticoid receptor antagonists

Mineralocorticoid receptor antagonists (MRAs) spironolactone and eplerenone exert similar cardioprotective effects [eplerenone showcasing slightly more pronounced cardioprotection (158)], however, they differ in their structure, selectivity, duration of action, side effects and therapeutic indications. Besides all these differences, their effect on glycemic control and T2DM prognosis remains elusive. Only a few small clinical trials have been conducted to study their effect on glycemic control with inconsistent and inconclusive results. Spironolactone showed increased HbA1c in hypertensive patients with T2DM (159) and with HF patients (160). Eplerenone has presumably no effect on HbA1c (160–162), blood glucose levels (163) and insulin resistance It does not increase the risk of triggering new onset of T2DM (164) and it doesn’t increase the need for antidiabetics (161). Its cardioprotective effect is independent of the presence of T2DM (163). In the case of the head-to-head comparison, one study (160) observed eplerenone as superior, however, another study did not find any strong evidence for eplerenone supremacy over spironolactone in the context of glycemic control (161). The guidelines (5) don’t provide any information about the preferred MRAs in patients with DCM.

A new MRA, finerenone, was found to express some cardioprotective effects in patients with chronic kidney disease (CKD) and T2DM (165–167), although it is currently indicated for CKD in T2DM only. Therefore, conducting bigger trials to screen for its effects on HF or glycemia is essential for follow-up research.

#### 4.2.4 ARNI

ARNI is a fixed combination of angiotensin receptor type 1 antagonist valsartan with neprilysin inhibitor sacubitril. Neprilysin is a proteolytic enzyme degrading several vasoactive peptides, most notably atrial, brain, C-type natriuretic peptides (ANP, BNP, CNP) and others, including bradykinin. Moreover, neprilysin degrades endothelin-1 and it is responsible for the conversion of Ang II to vasoprotective Ang (1-7). Thus, sacubitril monotherapy was not satisfactory and valsartan is used to block undesirable RAAS activation (168).

ARNI has been shown to have a better outcome on morbidity and mortality compared to enalapril with or without diabetes (138). One smaller study (169) (12 participants) showed increased postprandial glycemia with ARNI compared to placebo which contradicts a huge PARADIGM-HF trial (170). In a *post-hoc* analysis, they managed to find a significant and persistent decrease in HbA1c compared to enalapril in the course of 3 years. In addition, ARNI decreased the need for the administration of new oral antidiabetics and insulin in these patients. This superiority of ARNI over ACE inhibitor enalapril could be described by its complex mechanism of action not only on heart, but also by several pleiotropic (vascular, renal and metabolic) effects. Of particular interest, ARNI provides a beneficial effect on the development of T2DM, affecting increased incretin levels, improved insulin sensitivity, lipolysis and lipid oxidation (168).

#### 4.2.5 Angiotensin receptor blockers

Angiotensin receptor blockers (ARBs) are used only when ACEi or ARNI are not tolerated (5) because of insufficient data regarding their effects on morbidity and mortality. Despite the fact, various ARBs (171–173) have been proven to be a safe alternative for HF patients with or without diabetes.

#### 4.2.6 Ivabradine

Ivabradine is indicated for patients with sinus rhythm (SR) who do not tolerate betablockers or have an unsatisfactory response to betablocker therapy (5). Ivabradine seems to be safe in HF patients with T2DM and its effect is independent of the presence of T2DM (146, 174).

#### 4.2.7 Digoxin

Digoxin may be considered the drug of choice to reduce the risk of hospitalization in patients with HFrEF with SR when standard 1st-line therapy is insufficient with or without present T2DM (5). Data on the negative effects of digoxin on glucose tolerance are limited to small studies and case reports (175, 176). On the other hand, larger trial with 1933 patients in diabetic group showed no detrimental effects on HF-related hospitalizations compared to non-diabetic group, although it had no effect on overall mortality in both groups and diabetic patients experienced more frequently digoxin-toxicity-related hospitalizations. HbA1c or FPG values were not tested during this trial (177). Digoxin has also been used in HF patients with atrial fibrillation (AF) which is the official therapeutical indication, even though its safety in these patients is even more questionable and it should be used only if other therapeutical options are not available (71).

#### 4.2.8 Vericiguat

Vericiguat, a soluble guanylate cyclase (sGC) inhibitor, is a novel drug in the array of anti-HF agents. It has also been studied in T2DM patients (178), nonetheless, its effect on glycemic control remains unknown.

#### 4.2.9 Mavacamten

Mavacamten is another novel drug acting as myosin inhibitor and is indicated for the treatment of cardiac hypertrophy. Although there were diabetic patients included in the trials (179), no glycemic studies were conducted.

### 4.3 Adjuvant therapy

#### 4.3.1 Diuretics

Although there is not enough evidence for the use of diuretics in the prevention of CV events in patients with HF, they are still used to alleviate the sign of fluid congestion (5). Thiazide diuretics include several agents from which hydrochlorothiazide (HCT) is the most frequently used. HCT has been linked to a negative metabolic effects such as increased fasting plasma glucose (FPG), HbA1c, low-density lipoprotein-cholesterol (LDL-C) (180), unlike indapamide (181). Despite that, HCT cannot be substituted with indapamide due to its practically zero diuretic effects (HCT effects are more pronounced in blood vessels, rather than enhancing diuresis) (182). Another thiazide, chlorthalidone, may represent an alternative with better metabolic profile (183), although it has not been specifically tested for the treatment of HF.

Loop diuretics, such as furosemide, exert more potent diuretic effect. Similarly to HCT, they can cause glucose homeostasis impairment with reduced tissue sensitivity to insulin (184). The mechanism is independent of its diuretic properties as it solely relies on off-target tissue mechanisms. They supposedly interfere with basal tissue glycolysis by direct and indirect effects on glycolytic enzymes (185, 186) which is a shared mechanism with HCT. The use of loop diuretics in diabetic patients was linked to a negative impact on morbidity and mortality with worse impact on HF patients. SGLT2i, such as empagliflozin, are similarly effective in the alleviation of congestive symptoms in HF with better clinical and safety results (187).

#### 4.3.2 Lipid-lowering therapy

Statins are indicated in patients with combined dyslipidemia and hyperglycemia. They are generally well tolerated, except of their negative effects on glycemic control and liver toxicity. A meta-analysis from 2018 of 23 smaller and intermediate clinical studies (groups of 8-167 patients) showed differential effects of statins on the glycemic control. More favorable effect on FPG and HbA1c have pitavastatin (2mg), simvastatin (10-40 mg) and fluvastatin (20-40 mg). The greatest negative impact on glycemic control was observed in atorvastatin 80 mg, atorvastatin 10-40 mg and rosuvastatin 10-40mg. Pravastatin (10-20 mg) was in-between (188). Neutral glycemic effect was also observed with lovastatin (40 mg) (189). Needless to say, these differences do not correlate with their clinical efficacy in lowering LDL-C and overall effect on CV hospitalizations. High-intensity statins (atorvastatin, rosuvastatin) are used in patients with high CV risk as they lower LDL-C by 40–63% and significantly reduce the incidence of major cerebral and coronary complications (190). This beneficial effect outweighs the potential diabetogenic effect of these drugs, estimated as a 9% increased risk of incident diabetes, especially in older patients and in patients that are already at risk of developing diabetes (191, 192).

In patients with inadequate LDL-C control, ezetimibe can be added to statin. Ezetimibe does not impact FPG or HbA1c and ezetimibe with low-dose statin compared with high-dose statin therapy may have a beneficial tendency of effects on glycemic control (193). In resistant hypercholesterolemia, proprotein convertase subtilisin/kexin type 9 (PCSK9) inhibitors (evolocumab and alirocumab) are effective and safe choice, hence they do not alter glucose homeostasis and they do not trigger new onset of diabetes (194–196). Although, some Mendelian randomization analyses concomitant with post-marketing monitoring reports evidence of mild increase of hyperglycemia, rather than diabetes, in the first 6 months of the therapy with PCSK9 inhibitors (197).

Inclisiran is indicated for patients not tolerating PCSK9 inhibitors with the mechanism similar to that of PCSK9 inhibitors, except it acts as a gene-silencing therapy. As inclisiran is still a new drug, there is a lack of data concerning its effect on glycemic control and the possibility of new-onset T2DM cannot be completely ruled out (197). Patients with good lipid control but intolerating statins can be treated with bembedoic acid. Bembedoic acid is another novel hypolipidemic drug inhibiting cholesterol synthesis. Apart from statins, its bioactivation occurs mainly in liver (practically not in muscles) and it significantly reduces myalgia (198) and the new onset or worsening T2DM risk (199, 200).

PPAR-α agonists (fenofibrate, bezafibrate, clofibrate) are indicated for patients with hypertriglyceridemia. Their use is, however, limited due to their hepato- and nephrotoxic potential with low clinical benefit in randomized clinical trials (RCTs), aside from patients with very high triglyceride levels. Pemafibrate is a novel PPAR-α agonist with higher efficacy, and it has shown no clinically adverse effects on renal or hepatic function (5, 201).

## 5 Ongoing research and future therapy

Current guidelines and medications for the management of DCM are based on treating T2DM and HF separately, as there is no medication indicated specifically for the treatment of DCM. Additionally, the impact of the present pharmacotherapy is unsatisfactory with only little effect on overall progress of the disease and subsequent prognosis. This might change in near future as new medications are being developed and tested. Here we provide a brief summary of some interesting and promising therapeutic tools and targets.

### 5.1 Experimental drugs and approaches for the treatment of DCM

Novel pharmaceuticals need to provide a unique approach for treating DCM. The main targets of recent development are cardiac metabolism, oxidative stress, inflammation and cardiac fibrosis and hypertrophy. As currently available drugs do not provide required efficiency the research is very important. The list of experimental drugs is described in **Table 1**. Please note that most of the agents affect multiple pathological mechanisms (or one mechanism affects the other), thus we focus on their main therapeutic goal.

**Table 1:** An overview of experimental therapies in various models of DCM.

Primary beneficial effect	Agent	Molecular target (pharmacodynamics)	Model	References
Cardiac metabolism	caficrestat (AT-001)	aldose reductase (inhibitor)	Phase 3 RCT	(202)
	cemtirestat		fructose/STZ diabetic rats	(203)
	ninerafaxstat (IMB-1018972)	3-ketoacyl-CoA thiolase (inhibitor)	Phase 2 RCT	(204)
	rFGF21	FGF21 (recombinant)	STZ/high-fat diet diabetic mice	(205, 206)
	sulfo-N-succinimidyl oleate (SSO)	fatty acid translocase/cluster of differentiation 36 – FAT/CD36 (inhibitor)	STZ /high-fat diet diabetic rats	(207)
Oxidative stress	SIRT3 AAV	SIRT3 (adeno-associated virus vector)	STZ -induced diabetic mice	(208)
	mito-TEMPO	superoxide (antioxidant)	STZ -induced diabetic mice	(209)
	N-acetylcystein	glutathione peroxidase (precursor, antioxidant); PKCβ2 and eNOS signaling induction	STZ -induced diabetic mice and rats	(210–213)
Fibrosis and remodeling	H2-RLX, H3-RLX	relaxin (recombinant)	Ren2 hypertensive STZ -induced diabetic rats	(214, 215)
	FT23	TGF-β (inhibitor of unknown pharmacodynamics)	Ren2 hypertensive STZ -induced diabetic rats	(216)
	FT011	TGF-β (inhibitor of unknown pharmacodynamics)	Ren2 hypertensive STZ -induced diabetic rats	(217)
	gefitinib	EGFR (tyrosine kinase inhibitor)	STZ-induced diabetic mice	(218)
	Ang (1-7)	Mas (agonist)	STZ -induced diabetic rats	(219–221)
	DIZE	ACE2 (inhibitor)	db/db mice	(222)
	tadalafil	PDE5 (inhibitor)	Phase 4 RCT	(223)
	vardenafil	PDE5 (inhibitor)	ZDF rats	(224)
Inflammation	MCC950	NLRP3 (inhibitor)	high-sucrose and high-fat diet diabetic rats	(225)
	Fingolimod	S1P-R (functional antagonist)	STZ -induced diabetic mice and rats	(226–228)
	SB 203580	p38 MAPK (inhibitor)	STZ -induced diabetic mice	(229)
	pyrrolidine dithiocarbamate (PDTC)	NF-κB (inhibitor)	obese db/db diabetic mice	(230)

RCT, randomized clinical trial; ZDF, Zucker Diabetic Fatty; STZ, streptozotocin.

#### 5.1.1 Cardiac metabolism

The most promising new drug targeting cardiac metabolism that made it to the clinical trial is caficrestat (formerly known by its generic name AT-001). Caficrestat acts as an aldose reductase (AR) inhibitor. The physiological purpose of AR is to reduce glucose into sorbitol. During DCM, the expression of AR is increased which causes an exacerbation of various deleterious effects of DCM, such as ROS generation or fatty acid β-oxidation which in turn deteriorates energetic homeostasis of cardiac cells. Inhibition of AR by caficrestat improves diastolic function and decreases cardiac fibrosis and hypertrophy in patients with DCM (231). While the caficrestat is being investigated in phase 3 clinical trial (ARISE-HF, NCT04083339) (202), more efficient agents can potentially surpass it. Cemtirestat is a dual AR inhibitor with antioxidant activity. It is effective in fructose/STZ diabetic rats and serves as a good candidate for future clinical studies (203).

Another drug being tested in clinical trials is ninerafaxstat (IMB-1018972). Ninerafaxstat is 3-ketoacyl-CoA thiolase (acetyl-CoA acyltransferase) inhibitor. 3-ketoacyl-CoA thiolase is a catalyst of mitochondrial β-oxidation of long chain fatty acids, more specifically, it catalyzes the final step of β-oxidation in which 3-ketoacyl-CoA is cleaved by the thiol group of another molecule of coenzyme A (232). Ninerafaxstat is a cardiac mitotrope, it increases myocardial metabolic efficiency by shifting substrate utilization towards glucose through reduction in fatty acid oxidation (233). Its beneficial effect in clinical setup is being investigated in phase 2 clinical trial on cardiac energetics in T2DM and obesity with HFpEF (IMPROVE-DiCE trial, NCT04826159) (204).

Sulfo-N-succinimidyl oleate (SSO) is a lipid-derivative-based inhibitor of a membrane protein complex called fatty acid translocase/cluster of differentiation 36 (FAT/CD36). In the model of STZ/high-fat diet diabetic mice it decreases myocardial fatty acid oxidation rate and triglyceride concentration and increasing fatty acid metabolism, glycolytic rate and pyruvate dehydrogenase activity, improving cardiac function after hypoxia and reoxygenation (207). On the contrary, recombinant fibroblast growth factor 21 (FGF21) (205) or administered as a gene via adenovirus-associated virus (AAV) vector (206) in animal models with STZ/high-fat diet diabetic mice they both attenuated cardiac lipotoxicity by the activation of β-oxidation. As a result, it was accompanied by decreased ROS generation, inflammation, apoptosis and fibrosis. The proposed mechanism is complex, including AMPK (AMP-activated protein kinase)–Akt2–Nrf2-mediated antioxidative pathway and AMPK–ACC (acetyl CoA carboxylase)–CPT-1 (carnitine palmitoyl transferase 1)-mediated lipid-lowering effect in the heart. This mechanism is a little bit controversial, since cardiac β-oxidation inhibition [for instance by trimetazidine (234)] is a well-established cardioprotective mechanism in coronary artery disease. Thus, the cardioprotective effect of FGF21-induced β-oxidation activation could be rather caused by its effects on off-target organs, e.g., liver, where it controls the systemic lipid profile and metabolism (235). Moreover, the underlying cardioprotective mechanisms caused by FGF21 might not be fully understood and need more investigation, while no paper has discussed any overlap between FGF21 and trimetazidine or challenged this hypothesis so far.

#### 5.1.2 Cardiac oxidative stress

Pathophysiology of most cardiometabolic diseases involves increased ROS production and oxidative stress. Various antioxidants have been investigated in several models of cardiac injury which produced conflicting results (236). In the models of DCM, several agents attenuated cardiac oxidative stress, at least at a secondary level [e.g. pyrrolidine dithiocarbamate (PDTC) (230), flavonoids (237–241), melatonin (242), capsaicin (243), sulforaphane (244–247), oxymatrine (248), rFGF21 (205, 206), MSCs (249, 250), vardenafil (224), MCC950 (225) and trimetazidine (251)]. Currently, there is no antioxidant indicated for any CV disease [except for dexrazoxane in anthracycline toxicity (252) which can produce severe adverse effects and its use is limited(ref)] due to few and small clinical trials which were not necessarily following the results from preclinical studies.

To include some innovative approaches, SIRT3 gene administration as AAV in STZ-diabetic mice was studied mainly for its antioxidant properties leading to alleviation of mitochondrial dysfunction via inducing AMPK/FGF21/SIRT3 signaling axis (208). True chemical antioxidant properties possesses an experimental drug mito-TEMPO (209) and a mucolytic agent N-acetylcystein (NAC) (210–213). They both were able to decrease fibrosis and hypertrophy with improvement of systolic and diastolic contractile function as a result of decreased mitochondrial ROS.

#### 5.1.3 Cardiac fibrosis and remodeling

Fibrosis, remodeling and hypertrophy are the most crucial pathomechanisms within HF (39, 40). Their progress can be slowed down by ACEi, ARB, MRA, SGLT2i (5) or mavacamten (179), although not completely stopped or even reversed. Among the novel experimental drugs, recombinant relaxin [H2-RLX (214) and H3RLX (215)] and orally active TGF-β inhibitors [FT23 (216) and FT011 (217)] in the model of Ren2 hypertensive STZ-induced diabetic rats decreased heart collagen deposition with improvement of diastolic function (as a consequence of reduced myocardial stiffness). H2/3-RLX’s antifibrotic effect is mediated by the induction of MMP-1 and MMP-13 and enzymatic degradation of collagens (214). Additionally, relaxins reduce activation of pro-apoptotic caspases and inflammation by the inhibition of NLRP3 inflammasome and decreased IL-1β and IL-18 levels (215). TGF-β plays a crucial role in the activation of fibroblasts and production of extracellular matrix (253). These experimental drugs could be a game-changers because of their potent antifibrotic activity and high perspective as candidates for clinical trials in humans.

There is growing evidence showing the role of epidermal growth factor receptor (EGFR) family (including tyrosine kinase receptors, such as EGFR/ErbB/HER) in the development of DCM. EGFR has been linked to cardiac fibrosis, remodeling, hypertrophy, oxidative stress and inflammation (254). A selective EGFR inhibitor (gefitinib) decreased oxidative stress and attenuated diabetes-induced myocardial collagen deposition and fibrosis in STZ-induced diabetic mice. Moreover, it improved Ca^2+^ homeostasis and contractility by preventing the depletion of SERCA2a and NCX1 (sodium-calcium exchanger-1) (218). However, it seems that EGFR has a dual activity in CV morbidities. Despite being detrimental in the chronic setup, EGFR exerts pro-survival effects on acute myocardial injury (254, 255) and more advanced research on this topic is fairly required.

Alternative renin-angiotensin system (RAS) pathway - ACE2/Ang (1-7)/Mas axis plays a cardioprotective role in several CV morbidities and is gradually gaining more interest. ACE2/Ang (1-7)/Mas axis is often called a cardioprotective counterpart of canonical RAS. A sudden peak of attention was observed during COVID-19 pandemic because of SARS-CoV-2 viral particles using ACE2 to enter host cells. ACE2/Ang (1-7)/Mas axis counteracts Ang II by inducing vasodilatation and decreasing vascular/cardiac oxidative stress, inflammation, and fibrosis. Simultaneously, it can be downregulated in numerous hypoxic CV pathologies (256). Administration of exogenous Ang (1-7) peptide (or its analogue AVE-0991) improved vascular tone responsiveness, cardiac contractility and decreased oxidative stress in diabetic rats, while the inhibition of Ang (1-7) formation by ACE2 inhibitor DX600 or blockade of its active site by Mas receptor antagonist A779 deteriorated the cardiomyopathy (257–259). ACE2 activator diminazene aceturate (DIZE) was examined in db/db mice for its effect on DCM. DIZE was able to significantly attenuate cardiac fibrosis, remodeling and hypertrophy. In addition, this effect was accompanied by decreased oxidative stress and improved cardiac contractility (222). Similar results were observed after exogenous Ang (1-7) administration (219–221) with additive effect in the combination with perindopril (41). Moreover, direct antidiabetic effects have been observed as well. Pancreatic ACE2 gene therapy prevented the loss of β-cells, increased insulin production and improved fasting glycemia with no impact on insulin sensitivity (260), thus ACE2/Ang (1-7)/Mas axis acts as a pro-survival compensatory mechanism during DCM.

PDE5 inhibitors have been used as drugs for erectile dysfunction or pulmonary hypertension for many years now (261). Recently, tadalafil (20 mg once daily for 20 weeks) was investigated for its vasodilatory effect in diabetic hearts (RECOGITO trial, NCT01803828). It was successful in decreasing heart remodeling and inflammation with improved renal function, however, this effect was only observed in male participants, unlike their female counterparts (223). Additional beneficial results (decreased cardiomyocyte apoptosis, oxidative stress, myocardial hypertrophy and fibrosis with improved diastolic function) were observed in Zucker Diabetic Fatty (ZDF) rats treated with vardenafil l (224).

Some other agents are able to mitigate fibrosis via their primary target and include caficrestat (231), mito-TEMPO (209), fingolimod (226), NAC (211–213), MSCs (250, 262), melatonin (242), flavonoids (237), capsaicin (243) and sulforaphane (244–247).

#### 5.1.4 Cardiac inflammation

To this date, there is currently no medication approved specifically for the treatment of myocardial inflammation. Few papers focused on this issue in DCM models trying to solve this problem. p38 MAPK inhibitor SB 203580 might have decreased cardiac inflammation but did not change cardiac fibrosis and diastolic dysfunction compared to placebo (229). Better results were obtained with NLRP3 inflammasome inhibitor MCC950 which was able to ameliorate cardiac inflammation with additional decrease in apoptosis, oxidative stress and fibrosis (225). Some positive effects were also found with fingolimod treatment which promoted improved myocardial blood perfusion, contractility and decreased fibrosis (226–228). Pyrrolidine dithiocarbamate (PDTC) acting as an inhibitor of pro-inflammatory NF-κB in obese db/db diabetic mice mitigated oxidative stress, improved mitochondrial structural integrity, increased ATP synthesis and ultimately restored cardiac function (230).

H3-RLX (215), FT23 (216), FT011 (217), AAV FGF21 (206), tadalafil (223), MSCs (249, 250), oxymatrine (248), flavonoids (239), capsaicin (243), ginsenosides (263) and sulforaphane (244–247) also exert anti-inflammatory properties as a secondary effect.

Nevertheless, none of these studies looked at overall systematic side effects with the concern for general immunosuppression and negative CV side effects observed with many common immunosuppressants (264). Ultimately, small studies (13-50 patients per group) found etanercept (265–267), methotrexate (268) and thalidomide (269) safe and effective for improving quality of life. On the other hand, larger studies (270) (>300 patients per group) did not find any significant improvement of immunosuppression in HF. Clinical benefit of prednisone and azathioprine in dilated cardiomyopathy with possible myocarditis is similarly controversial (271, 272).

#### 5.1.5 Cardiac cell death

Necroptosis poses a novel approach in the understanding of cardiomyocyte cell death. As we mentioned above (22, 23), RIPK3 and CaMKII may play an important role in the pathophysiology of DCM. Although there are several studies of RIPK3 inhibition (GSK’872) during I/R injury (273) and cardiac fibrosis (274)/hypertrophy (275) via CaMKII pathway, there are no record on DCM yet. Ultimately, apoptosis and necroptosis are under the physiological conditions the last resort of defense against cancer (276) and viral infections (277, 278), thus systemic side effects of their inhibition cannot be underestimated and must be taken with precaution.

#### 5.1.6 Dietary and herbal therapeutics for patients with DCM

Among the numerous experimental drugs for DCM, there are also some experimental studies involving several natural products (see **Table 2**). Ginsenosides from plant *Panax ginseng* can inhibit ROS production, stimulate NO production, increase blood circulation, ameliorate vasomotor tone and adjust lipid profile (280). In the animal model of DCM, ginsenosides were observed to decrease cardiac inflammation via inflammasome and pyrroptosis inhibition (263). *Sophora flavescens*, a plant from Chinese medicine, contains a quinolizidine alkaloid oxymatrine possessing anti-inflammatory, antifibrotic and antioxidant properties (248, 279, 281) in models of HF, I/R injury and DCM. Flavonoid compounds are natural plant antioxidants (282) found ubiquitously in the plant kingdom. Quercetin (237, 238), apigenin (239), dihydromyricetin (240) and naringenin (241) were identified to decrease oxidative stress and inflammation in animal models of DCM which manifested as improved contractility and decreased cardiac fibrosis. Within the flavonoid chemical group, diosmin, hesperidin and oxerutin (283, 284) are already well-established compounds in the treatment of various circulatory diseases, such as hemorrhoids, chronic venous insufficiency and others. Increasing evidence suggests that cardioprotective effects of flavonoids in various cardiometabolic diseases should not be underestimated as they propose good candidates for bigger clinical trials targeting not only the blood vessels but also the heart.

**Table 2:** Herbal and dietary components in the treatment of DCM.

Natural product	Mechanism	Plant of origin	References
ginsenosides	various	*Panax ginseng*	(263)
oxymatrine	various/unknown	*Sophora flavescens*	(279)
capsaicin	TRPV1 (agonist/activator)	*Capsicum annuum*	(243)
flavonoids (quercetin, apigenin, dihydromyricetin, naringenin…)	various	various	(237–241)
sulforaphane	Nrf2 (activator)	*Brassica oleracea* (broccoli, brussels sprouts, cabbage…)	(244–247)
melatonin	free radical scavenger and others	ubiquitous (animals, plants, fungi…)	(242)

Besides herbal medicine, common dietary components were also found to improve symptoms of DCM. The main and best-known spicy constituent of peppers (*Capsicum annuum*), capsaicin, activates not only sensory but also vascular and cardiac TRPV1 ion channels and modulates multiple pathological processes in diabetic hearts (243). Cultivars of *Brassica oleracea* (broccoli, brussels sprouts, cabbage, etc.) are known to decrease cardiac oxidative stress, hypertrophy, and fibrosis with increase of systolic function through their metabolite sulforaphane, a Nrf2 activator (244–247). Melatonin was first discovered in animals but later also found in plants, fungi, and bacteria. It has a profound antioxidant effects acting primary as a free radical scavenger, while it also stimulates antioxidative enzymes, increases the efficiency of mitochondrial oxidative phosphorylation and reduces electron leakage thus lowering free radical generation (285). Beyond the effects on OS, mitochondria and myocardial cell metabolism, melatonin reduces vascular endothelial cell death, reverses microcirculation disorders, reduces myocardial fibrosis and regulates cell autophagy and apoptosis (242).

### 5.2 Future therapeutic promises

Based on current and experimental therapeutic options for DCM discussed in this review, it is obvious that extensive research is yet to come. In fact, treatment itself will not affect the disease prognosis in most cases. It is based on improving the quality of life and postponing hospitalization for a relatively brief period, thus only symptomatic, not causal. The main reason is a poor understanding of underlying pathophysiology of DCM. Fibrosis is one the main drivers of HF (39), though it can be only slowed down, not reversed. Fibrosis is accompanied by chronic inflammation (39) but again, current (nor experimental) drugs are not very efficient or selective in its treating. Finally, the cell death of cardiomyocytes is almost not addressed at all. Although some experimental drugs already exist, they are not selective for cardiac tissue and can be very toxic (e.g., risk of cancer) (286).

The answer (for at least maximizing efficacy with limited toxicity) can be using specialized drug delivery systems (287–289), such as nanopolymers, lipid-based nanocarriers, cyclodextrins and gene delivery. This might be a game changer in treating HF; nevertheless, massive research is still to be done. It is theoretically plausible even to reverse the damage of the heart. There is some evidence for MSCs and their exosomes to be able to restore pancreatic β-cells (290) or to replace dead cardiomyocytes (262), although this mechanism has become controversial in recent years (291) (injected stem cells do not survive for a long period of time and paracrine regulation is becoming a more popular theory). Therefore, the real cure for DCM lies ahead.

## Author contributions

PG: Visualization, Investigation, Writing – review & editing, Writing – original draft, Validation, Conceptualization. LB: Visualization, Writing – review & editing, Writing – original draft, Validation. VF: Writing – review & editing, Writing – original draft, Validation, Funding acquisition. MB: Writing – review & editing, Writing – original draft, Validation, Funding acquisition. KF: Writing – review & editing, Writing – original draft, Validation. TR: Writing – review & editing, Writing – original draft, Validation, Supervision, Resources, Project administration, Funding acquisition, Conceptualization.
